# Complications in cesarean sections: A national survey of obstetric protocols and outcomes in Spain

**DOI:** 10.1371/journal.pone.0330352

**Published:** 2025-09-03

**Authors:** María Jesús Cancelo Hidalgo, José Eliseo Blanco-Carnero, Lucas Cerrillos González

**Affiliations:** 1 Department of Obstetrics and Gynaecology, Universitary Hospital of Guadalajara, Guadalajara, Spain; 2 University of Alcalá, Alcalá de Henares, Spain; 3 Department of Obstetrics and Gynecology, Virgen de la Arrixaca University Clinical Hospital, Murcia, Spain; 4 Biomedical Research Institute of Murcia Pascual Parrilla–IMIB, Murcia, Spain; 5 Maternal-Fetal Medicine, Genetics, and Reproduction Unit. Virgen del Rocío University Hospital, Seville, Spain; Kasr Alainy Medical School, Cairo University, EGYPT

## Abstract

**Introduction:**

Cesarean sections are among the most common obstetric surgeries worldwide. While generally safe, they can be complicated by numerous factors increasing risks for both mother and fetus, and posing significant challenges in clinical practice. In Spain, the absence of unified protocols for managing high-risk cases underscores the need for systematic guidance to improve maternal outcomes and reduce morbidity.

**Materials and methods:**

A 45-question survey evaluated the management of complicated cesarean sections in Spain among members of the Perinatal Medicine Section of the Spanish Society of Gynecology and Obstetrics. The survey was developed and internally validated by the Spanish Society of Gynecology and Obstetrics. It was distributed online to all registered members. A total of 744 responses were collected between March and June 2024. Inclusion criteria were current obstetric practice and at least one year of experience.

**Results:**

Data from 744 Spanish gynecologists was gathered. Respondents, had a median of 15.0 (P25-P75 = 17.0–24.0) years of experience, reported performing an average of 43.4 (SD = 62.1) cesarean sections per month, with 21.9% classified as complicated. Hospital level influenced case volume and complexity, with higher-level hospitals reporting higher incidence of complicated cesarean sections. Only 14.5% of institutions had established preoperative protocols for complicated cesarean sections, showing improved outcomes when implemented. Key complications included blood loss (1000–1500 ml in 35.3% of cases), fetal extraction difficulties, uterine atony, and adjacent organ trauma. Postoperative issues such as infections and wound dehiscence were also common.

**Conclusion:**

This study highlights the need for standardized protocols in Spain to manage complicated cesarean sections. Their implementation could reduce intraoperative blood loss, decrease maternal morbidity from hemorrhage and trauma, and improve postoperative recovery and hospital stay duration.

## Introduction

Cesarean section (CS), defined as the birth of a fetus through a surgical incision on the abdominal wall (laparotomy) and uterine wall (hysterotomy), is among the most widely practiced obstetric surgical procedures worldwide [1]. Cesarean delivery has become a routine obstetric procedure, often regarded as a straightforward and low-risk alternative to natural childbirth, a perception likely influenced by the notable advancements in its safety since the mid-20th century [2,3]. However, in some instances, this surgical procedure can be technically difficult with consequent health hazards for both the mother and the fetus [4]. In recent decades, Spain has experienced a significant rise in cesarean section rates, increasing from 15% to 25% in the public healthcare system and from 28% to 38% in the private sector. This upward trend is attributed to a variety of contributing factors, many of which remain insufficiently defined or understood [5].

Complications during cesarean sections (CSs) may arise from challenges such as accessing the lower uterine segment, difficult fetal extraction, lacerations, organ damage, or abnormal placentation. These issues, along with uterine rupture and adhesions, present significant challenges in obstetric practice [6,7]. These complications can lead to increased maternal mortality and morbidity, as well as long-term gynecological conditions, including chronic pain, infertility, and pelvic adhesions [8].

Nevertheless, the demographic and clinical profiles of patients undergoing CSs are evolving, with an increase in factors such as advanced maternal age, high Body Mass Index (BMI), and repeated CS deliveries contributing to a rise in complicated cases [9]. Systematic protocols are essential for the anticipation and management of intraoperative complications in high-risk CSs [10]. These protocols provide structured guidance for clinicians to identify, prepare for, and mitigate potential risks, with a primary emphasis on reducing intraoperative blood loss, a significant contributor to maternal morbidity and mortality [11]. Comprehensive strategies typically encompass hemostasis and blood management, integrating risk assessment, quantitative blood loss measurement, early administration of uterotonic agents, and fixed-ratio transfusion protocols [12–14].

Therefore, the objective of this study is to explore current practices in the management of complicated CSs in Spain by describing prevailing clinical practices, identifying commonly perceived complications, and highlighting opportunities for optimization and standardization to improve early identification and management of high-risk cases. To address these challenges, the study presents results from a nationwide survey of members of the Spanish Society of Gynecology and Obstetrics (SEGO), capturing real-world practices and perceptions from a large and diverse sample of Spanish gynecologists across various hospital levels. This survey provides granular insights into variations in protocol availability, multidisciplinary collaboration, and complication management. To our knowledge, it is one of the first comprehensive studies to address these aspects at a national level in Spain, offering valuable data to inform future policy and guideline development tailored to the Spanish healthcare context.

## Materials and methods

### Study design and participants

This is a descriptive, cross-sectional, national survey to gather real-time data on current practices and complications associated with cesarean sections. The survey was distributed electronically through the official platform of the national scientific society, reaching all its registered members. The target group consisted of members of the Perinatal Medicine Working Group, dedicated to obstetric medicine. Participation was voluntary and anonymous, constituting a voluntary sampling method within a predefined population. Respondents self-selected based on interest or availability. To increase the response rate and minimize non-response bias, periodic reminders were sent via institutional email during the data collection period. 744 surveys were obtained from March to June 2024.

The participants were practicing gynecologists from various hospitals, stratified by hospital level, who were invited to complete a structured survey. Inclusion criteria required participants to be actively involved in obstetric care, with a minimum of one year of experience working as a gynecologist (asked in the survey). The study aimed to capture data representative of diverse clinical settings, ranging from small regional hospitals to large tertiary care centers. The study did not involve direct research on human subjects, nor did it include the collection or processing of personal or sensitive data.

Hospitals where respondents work were classified into four levels based on the annual number of deliveries. Level 1 included centers with fewer than 600 deliveries per year. Level 2 comprised hospitals with at least 600 but fewer than 1,200 deliveries annually. Level 3 encompassed hospitals with at least 1,200 but fewer than 2,400 deliveries per year. Finally, Level 4 consisted of hospitals with 2,400 or more annual deliveries. No further stratification was performed.

### Survey development

A structured survey was designed to gather detailed information about gynecologists’ practices and perceptions regarding complicated cesarean sections. The tool was developed with the aim of capturing a comprehensive and multidimensional view of this clinical scenario, covering key areas such as case definition, frequency, protocol implementation, and clinical decision-making. The survey was administered in Spanish with an English translation provided in Annex 1 for reference. The survey included 45 items and was divided into the following sections: 1) Demographic and Professional Information: Questions about age, gender, years of experience, country, and type and level of the hospital where participants practiced. 2) Definition and Frequency of Complicated Cesarean Sections: Items exploring participants’ definitions, the prevalence of such cases in their practice, and the frequency of pre-identified complicated cesareans. 3) Preoperative Protocols and Decision-Making: Questions on the availability of written protocols, diagnostic tests, involvement of other specialists, and strategies for preoperative planning. 4) Intraoperative and Postoperative Management: Items addressing surgical techniques, blood loss, complication rates, and postoperative care practices. 5) Complications and Training: Questions about the most common complications encountered, training on simulators, and adherence to evidence-based protocols such as ERAS (Enhance Recovery After Surgery)/ACIERTO (*Asistencia de Calidad Integral y Estándar para una Recuperación Total Óptima*).

### Types of questions

The survey included a mix of multiple-choice, ranking, and open-ended questions. For example, participants were asked to rank factors contributing to complications during fetal extraction, such as deep head impaction or fetal macrosomia, and to identify diagnostic tests used for suspected complicated cases, such as imaging or pre-anesthetic evaluations. Multiple-choice questions covered the frequency of complications and the presence of written protocols, while open-ended items allowed participants to elaborate on their definitions of complicated cesarean sections. This question was included not to establish a formal definition, but to describe how this concept is currently interpreted in clinical practice across different settings.

### Data collection

The survey was distributed electronically using a secure online platform to ensure broad participation and maintain confidentiality. Responses were anonymized to protect participant identity, and no identifiable personal information was collected. Participants were informed about the study’s objectives and provided informed consent prior to completing the survey. The survey required approximately 10 minutes to complete.

### Statistical analysis

Data were analyzed using descriptive and inferential statistics to summarize participant characteristics, identify common practices, and examine variability across hospital types and levels. For the statistical analysis, qualitative variables were summarized as absolute and relative frequencies, while quantitative variables were reported as mean and standard deviation or as median and interquartile range (IQR) in cases where normality was not met. The IQR was expressed as the range between the 25th and 75th percentiles. The normality assumption was assessed using the Shapiro-Wilk test. All statistical analyses were performed using SAS software, version 9.4.

## Results

### Characteristics of the respondents

The median age of respondents was 42.0 (P25-P75 = 35.0–52.0) (Table 1), with the highest median age observed in level 4 hospitals (44.5 years, P25-P75 = 35.0–55.0) (S1 Table). The majority of respondents were female (75.9%, n = 564) (Table 1), with gender distribution remaining relatively consistent across hospital levels. The highest percentages of male respondents were observed in level 4 and level 1 hospitals, accounting for 29.2% and 25.0% of participants, respectively (S1 Table).

**Table 1:** Characteristics of respondents in the national survey of obstetric protocols and outcomes in Spain (March-June 2024). n = 744.

Survey Question	Results
**Age, median years (P25-P75)** ^**1**^	42.0 (35.0–52.0)
**Gender, n (%)** ^**2**^	
Male	179 (24.1)
Female	564 (75.9)
**Experience as a gynecologist, median years (P25-P75)**	15.0 (7.0-24.0)
**Type of hospital, n (%)** ^**3**^	
Public	558 (75.2)
Private	100 (13.5)
Public-private	84 (11.3)
**Hospital level, n (%)** ^**3**^	
Level 1: Centers with less than 600 deliveries	124 (16.7)
Level 2: Centers with 600 deliveries or more and less than 1,200 deliveries.	176 (23.7)
Level 3: Centers with 1,200 deliveries or more and less than 2,400.	248 (33.4)
Level 4: Centers with ≥2,400 deliveries	195 (26.2)
**Cesarean sections per month, median (P25-P75)**	25.0 (15.0–49.5)
**Complicated cesarean sections per month, median (P25-P75)**	5.0 (2.0–10.0)
**Percentage of complicated cesarean sections per month, median (P25-P75)**	20.0 (10.0-28.6)

^1^Based on n = 722.

^2^Based on n = 743.

^3^Based on n = 743.

Respondents reported a median of 15.0 years of professional experience as gynecologists (P25-P75 = 7.0–24.0) (Table 1). Those employed in level 4 hospitals had a higher median of 18.0 years (P25-P75 = 10.0–25.0) (S1 Table).

In terms of the type of hospital, 75.2% (n = 558) of respondents worked in public institutions, 13.5% (n = 100) in private hospitals, and 11.3% (n = 84) in public-private centers (Table 1). As shown in S1 Table, participants from level 1 hospitals (n = 124) had a median age of 42.0 years (P25-P75 = 35.0–56.0) and the highest proportion of private-sector professionals (25.8%), with a median of 8 cesarean sections performed per month (P25-P75 = 5.0–12.0), of which only 1.0 (P25-P75 = 1.0–2.0) were classified as complicated. In level 2 hospitals (n = 176), the participant profile was similar in terms of age and gender, with 70.5% working in public institutions. The surgical volume was higher, with a median of 20 cesarean sections per month (P25-P75 = 14.0–30.0) and 4.0 (P25-P75 = 2.0–5.0) being complicated. Respondents from level 3 hospitals (n = 248) reported a median of 30 cesarean sections per month (P25-P75 = 20.0–40.0), with 5.0 (P25-P75 = 3.0–10.0) considered complicated, and the highest proportion of public hospital affiliation (77.4%). Level 4 hospitals (n = 195) included the oldest participants (median age 44.5 years, P25-P75 = 35.0–55.0) and the most experienced professionals (median 17.8 years). These respondents worked almost exclusively in public hospitals (85.6%) and reported the highest surgical volume, with a median of 60 cesarean sections per month (P25-P75 = 40.0–100.0) and 10.0 (P25-P75 = 5.0–20.0) classified as complicated.

Regarding the geographical distribution of responses, they were broadly distributed across autonomous communities. The most represented regions were Andalusia (17.9%), the Community of Madrid (17.1%), the Valencian Community (12.0%), and Catalonia (10.9%). Detailed percentages for all regions are provided in S1 Fig.

### Definition and frequency of complicated cesarean sections

The majority of respondents (80.1%, n = 596) described complicated CS as a surgery involving medical or surgical complexity, irrespective of whether complications occur postoperatively. A total of 59.8% (n = 445) defined it as a procedure associated with more complications than typically expected. 10.8% (n = 80) considered a complicated CS to be one that necessitates the involvement of a senior attending physician, and 1.3% (n = 10) provided alternative definitions (Table 2). The definition of a complicated CS was largely consistent across hospital levels, with the majority of respondents agreeing it involves medical or surgical complexity regardless of postoperative outcomes. However, respondents from level 4 hospitals were more likely to emphasize the involvement of a senior attending physician in complex cases (16.4%) compared to those from lower-level hospitals (S2 Table).

**Table 2:** Characteristics of complicated cesarean sections reported in the national survey of obstetric protocols and outcomes in Spain (March-June 2024). n = 744.

Survey Question	Results
**How would you define a complicated cesarean section, n (%)**	
A cesarean section that may involve medical or surgical complexity (even if there are no subsequent complications)	596 (80.1)
The one that after its performance had more complications than usual (expected or not)	445 (59.8)
One that is expected to be performed by a senior attending physician	80 (10.8)
Other definitions	10 (1.3)
**Record of complicated cesarean sections and postoperative complications of obstetric surgery, n (%)**	197 (26.5)
**Are the consequences of cesarean section given the importance they deserve? n (%)**	390 (52.4)
**Frequency of complicated cesarean sections performed, n (%)**	
Less than 20%	502 (67.5)
Between 20% and 30%	203 (27.3)
Between 30% and 40%	39 (5.2)
**Frequency of cesarean sections previously identified as potentially complicated, n (%)**	
Less than 25%	600 (80.6)
Between 25% and 50%	114 (15.3)
More than 50%	30 (4.0)
**Frequency of complicated cesarean sections not previously identified as potentially complicated, n (%)**	
1–5%	307 (41.3)
5–10%	261 (35.1)
10–15%	78 (10.5)
15–20%	98 (13.2)
**Type of incision chosen in a complicated cesarean section, n (%)**	
Pfannensitiel	552 (74.2)
Misgav-Ladach	160 (21.5)
Median laparotomy	31 (4.2)
Cherney incision	1 (0.1)

Respondents reported performing a median of 25.0 CSs per month (P25-P75 = 15.0–49.5), with a median of 5.0 (P25-P75 = 2.0–10.0) classified as complicated, representing a median of 20.0% (P25-P75 = 10.0–28.6) of all monthly CSs performed (Table 1). The volume of CSs varied by hospital level. Gynecologists working at level 4 hospitals reported the highest monthly median of CSs (60.0, P25-P75 = 40–100), with a median of 10.0 (P25-P75 = 5.0–20.0) classified as complicated, accounting for a median of 18.9% (P25-P75 = 10.0–31.3) of all CSs performed at this level. In contrast, respondents from level 1 hospitals reported a lower volume, with a median of 8.0 CSs per month (P25-P75 = 5.0–12.0), of which a median of 16.7% (P25-P75 = 10.0–25.0) were considered complicated (S1 Table).

In this study, 26.5% (n = 197) of respondents reported maintaining records of complicated CSs and postoperative complications related to obstetric surgeries (Table 2). When stratified by hospital level, the highest proportions of record-keeping were observed in level 1 (29.8%, n = 37) and level 4 hospitals (28.7%, n = 56) (S2 Table).

Additionally, 52.4% (n = 390) of respondents agreed that the consequences of CSs receive adequate attention, with the highest proportion of positive responses from level 3 hospitals (56%, n = 139), and the lowest from level 4 hospitals (48.2%, n = 94) (S2 Table).

The self-reported frequency of complicated CSs varied among respondents. The majority (67.5%, n = 502) estimated that complicated CSs accounted for <20% of their total CSs, while 27.3% (n = 203) reported a 20–30% complication rate, and 5.2% (n = 39) indicated that 30–40% of CSs were classified as complicated (Table 2). A trend was observed across hospital levels, with the proportion of respondents reporting <20% complicated CSs decreasing as hospital level increased. Specifically, 89.5% (n = 111) of respondents from level 1 hospitals reported a complication rate <20%, compared to 46.7% (n = 91) from level 4 hospitals. Conversely, 43.6% (n = 85) of level 4 hospital respondents indicated a 20–30% complication rate, and 9.7% (n = 19) reported 30–40% (S2 Table).

Regarding preoperative identification, 80.6% (n = 600) estimated that <25% of CSs could be recognized as potentially complicated before surgery, while 15.3% (n = 114) estimated this proportion at 25–50% (Table 2). However, 41.3% (n = 307) and 35.1% (n = 261) of respondents reported that 1–5% and 5–10% of ultimately complicated CSs were not identified as such preoperatively, respectively. Notably, level 4 hospitals exhibited a higher proportion of missed complicated CSs, with fewer respondents reporting a 1–5% misclassification rate (27.7%) and more reporting 10–15% (16.4%) or 15–20% (20.0%) (S2 Table), suggesting greater challenges in preoperative risk assessment at higher hospital levels.

### Protocols of action in complicated cesarean sections

Out of the 744 respondents, 14.5% (n = 108) reported that their hospital had a written protocol for preoperative planning in cases where a complicated CS was anticipated (Table 3). Among these hospitals, 66.7% (n = 72) reported improvements following the implementation of the protocol. In hospitals without a protocol, specific actions were reported for managing unexpected complications during CSs in 57.9% (n = 368) of cases, whereas hospitals with protocols demonstrated slightly higher adherence, with specific actions reported in 65.7% (n = 71) of cases (Table 3).

**Table 3:** Characteristics of the action protocols reported in the national survey of obstetric protocols and outcomes in Spain (March-June 2024). n = 744.

Survey Question	Results		
	Total (n = 744) n (%)	With protocol (n = 108) n (%)	Without protocol (n = 636) n (%)
**Written protocol for preoperative planning when a complicated cesarean section is anticipated**	–	108 (14.5)	–
**Actions in the event of a cesarean section that was not expected to be complicated**	439 (59.0)	71 (65.7)	368 (57.9)
**Session or committee in which cases are presented and strategies are developed**	437 (58.7)	87 (80.6)	350 (55.0)
**Specialists decide who will be involved in an anticipated complicated cesarean section**			
Yes. Two attending physicians are required	387 (52.0)	56 (51.9)	331 (52.0)
It depends on the reason for the complexity	238 (32.0)	52 (38.9)	196 (30.8)
We do not decide who participates in the cesarean section	87 (11.7)	6 (5.6)	81 (12.7)
Yes. Two attending physicians are not required	32 (4.3)	4 (3.7)	28 (4.4)
**Prior contact with other specialists to evaluate the case**			
No contact with other specialists	321 (43.1)	30 (27.8)	291 (45.8)
General surgery	246 (33.1)	50 (46.3)	196 (30.8)
Urology	230 (30.9)	57 (52.8)	173 (27.2)
Interventional radiology	183 (24.6)	42 (38.9)	141 (22.2)
Depending on the case	54 (7.3)	2 (1.9)	52 (8.2)
Anesthesiologist	25 (3.4)	7 (6.5)	18 (2.8)
Other	50 (6.7)	7 (6.5)	43 (6.8)
**Actions implemented when a complicated cesarean section is suspected**			
Blood products reserve	712 (95.7)	106 (98.1)	606 (95.3)
Pre-anesthesia consultation	644 (86.6)	105 (97.2)	539 (84.7)
Consultation with other specialists	375 (50.4)	79 (73.1%)	296 (46.5)
Diagnostic imaging methods	374 (50.3)	72 (66.7)	302 (47.5)
Other	12 (1.6)	0 (0.0)	12 (1.9)
**Planned blood reserve**	733 (98.5)	107 (99.1)	626 (98.4)
**Pre-training in complicated extractions with simulators**	187 (25.1)	39 (36.1)	148 (23.3)
**Improvements observed since implementation of the written protocol**	–	72 (66.7)	–

Formal case review sessions or committees to discuss complicated CSs and develop management strategies were reported by 58.7% (n = 437) of respondents. These sessions were more frequently held in hospitals with protocols (80.6%, n = 87) compared to those without protocols (55.0%, n = 350). Regarding specialist involvement, respondents indicated that hospitals with protocols demonstrated higher collaboration with general surgeons (46.3%), urologists (52.8%), and interventional radiologists (38.9%) than hospitals without protocols, where collaboration rates were 30.8%, 27.2%, and 22.2%, respectively (Table 3).

Regarding the actions implemented when a complicated CS is suspected, 98.5% (n = 733) of respondents reported that their hospitals plan blood product reserves. When comparing hospitals with and without a written protocol for complicated CSs, respondents reported comparable rates of planning (99.1% and 98.4%, respectively). Regarding pre-anesthesia consultations, these were conducted in 86.6% (n = 644) of cases overall, with a higher frequency reported by respondents from hospitals with established protocols (97.2%, n = 105) compared to those from hospitals without protocols (84.7%, n = 539). Diagnostic imaging methods were utilized by 50.3% (n = 374) of respondents, with higher usage observed in hospitals with protocols (66.7%, n = 72) compared to those without (47.5%, n = 302). Pre-training with simulators for complicated CSs was reported by 25.1% (n = 187) of respondents, with this practice being more prevalent in hospitals with protocols (36.1%, n = 39) than in those without (23.3%, n = 148) (Table 3).

The availability of preoperative protocols for managing anticipated complicated CSs was more frequently reported in level 4 hospitals (22.6%) compared to level 1, level 2, and level 3 hospitals (12.9%, 10.2%, and 12.1%, respectively). The data on the prevalence of multidisciplinary case review sessions, specialist involvement, and simulator-based training for complex CSs across hospital levels among facilities with preoperative protocols are detailed in S3 Table.

### Intraoperative complications

In terms of intraoperative blood loss during complicated CSs, 7.9% (n = 59) of respondents reported a blood loss of less than 500 ml, while the majority (50.9%, n = 379) estimated blood loss between 500–1000 ml. Significant hemorrhage, defined as blood loss between 1000–1500 ml, was reported by 35.3% (n = 263) of respondents, and extreme blood loss (>1500 ml) occurred in 5.8% (n = 43) of cases (Table 4).

**Table 4:** Intraoperative and postoperative complications of cesarean section reported in the national survey of obstetric protocols and outcomes in Spain (March-June 2024). n = 744.

Survey Question	Results
**Blood lost during a complicated cesarean section, n (%)**	
<500 ml	59 (7.9)
500–1000 ml	379 (50.9)
1000–500 ml	263 (35.3)
>1500 ml	43 (5.8)
**Other than hemorrhage, what other immediate complications are associated with a cesarean section in your practice? n (%)**	
Difficult extraction of the fetus	675 (90.7)
Postpartum atony	635 (85.3)
Uterine tears	594 (79.8)
Trauma to adjacent organs	343 (46.1)
Other	15 (2.0)
**How often do complications arise in your hospital during cesarean section due to laceration or trauma to other organs? n (%)**	
Urological injuries	
Exceptional	394 (53.0)
0.5–1%	276 (37.1)
1–2%	66 (8.9)
>2%	8 (1.1)
Gastrointestinal lesions	
Exceptional	691 (92.9)
0.5–1%	45 (6.0)
1–2%	8 (1.1)
>2%	0 (0.0)
**Complications after cesarean section, n (%)**	
Surgical wound infections after cesarean section (n = 683)	
<3%	489 (71.6)
3–10%	179 (26.2)
10–15%	15 (2.2)
Endometritis infections after cesarean section (n = 670)	
<1%	475 (70.9)
1–3%	170 (25.4)
3–5%	25 (3.7)
Complications due to dehiscence after cesarean section (n = 656)	
<5%	588 (89.6)
5–10%	65 (9.9)
10–20%	3 (0.5)

Blood loss between 500–1000 ml was the most commonly reported range across all levels, with little variation; however, level 4 hospitals showed a slightly higher incidence of blood loss exceeding 1500 ml (7.2%) compared to level 1 hospitals (2.4%) (S4 Table).

Beyond hemorrhage, other intraoperative complications were frequently observed. The most commonly reported complication was difficult fetal extraction (90.7%, n = 675), followed by postpartum uterine atony (85.3%, n = 635) and uterine tears (79.8%, n = 594). Trauma to adjacent organs was reported by 46.1% (n = 343) of respondents. Urological injuries were predominantly considered exceptional by 53.0% (n = 394) of respondents, while 37.1% (n = 276) estimated a urological injury rate of 0.5–1%. Gastrointestinal injuries were significantly rarer, with 92.9% (n = 691) of respondents classifying them as exceptional and 6.0% (n = 45) reporting an injury rate of 0.5–1% (Table 4).

Regarding hospital levels, difficult fetal extraction and postpartum uterine atony were consistently reported across all levels. However, uterine tears and trauma to adjacent organs appeared to be reported slightly more frequently in higher-level hospitals.

**Fig 1:**
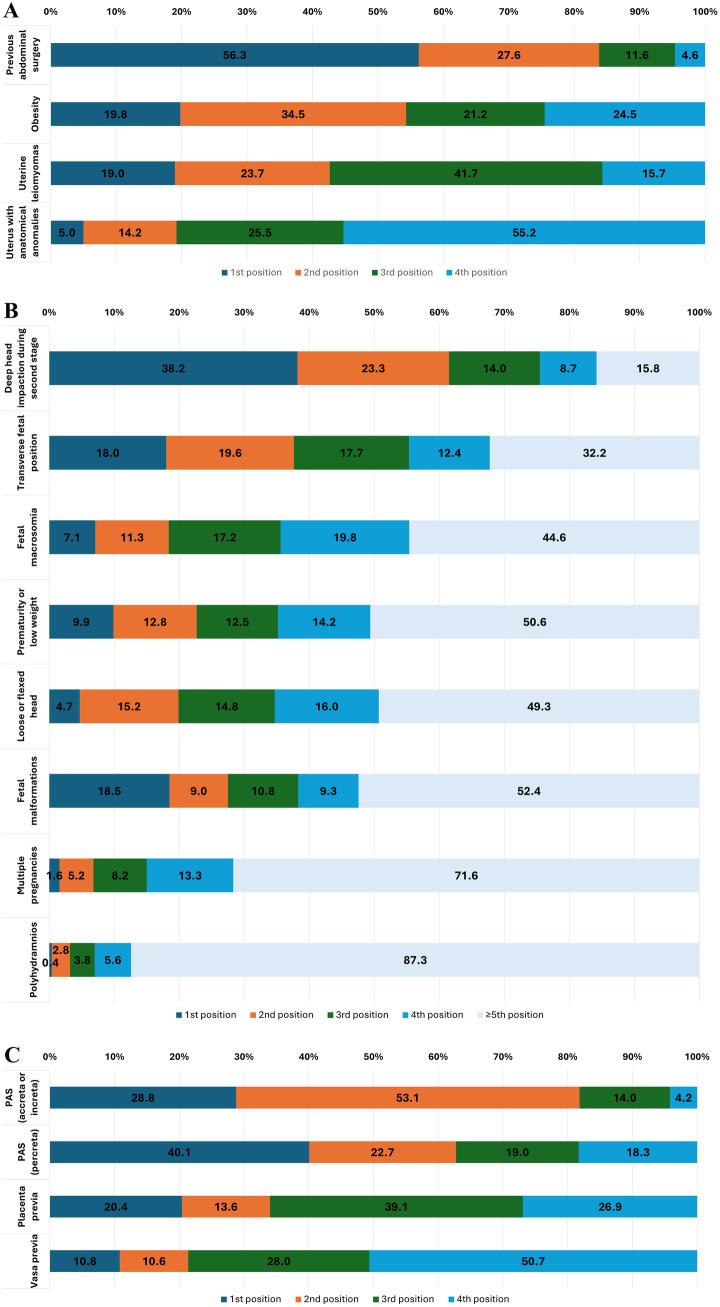
Ranking of factors in order of importance as reported by survey respondents regarding the difficulty in accessing the lower uterine segment (A), complicated fetal extraction in cesarean section (B), and anomalous placentation (C), stratified by hospital level. The rankings are represented as percentages for each factor. PAS: placenta accreta spectrum.

The factors contributing to complications during CS due to difficulty accessing the lower uterine segment were ranked by respondents in the following order of importance (from most to least important): previous abdominal surgery, obesity, uterine leiomyomas, and uterus with anatomical anomalies (Fig 1A). Similarly, the factors impacting complications during complicated fetal extraction CS were ranked as follows (from most to least important): deep head impaction during the second stage, transverse fetal position, fetal macrosomia, prematurity or low weight, loose or flexed head, fetal malformations (e.g., hydrops fetalis, tumors, conjoined twins), multiple pregnancies, and polyhydramnios (excess amniotic fluid) (Fig 1B). Finally, the complication factors for CS due to anomalous placentation were ranked as follows (from most to least important): placenta accreta spectrum (PAS), placenta previa, and vasa previa (Fig 1C). The data illustrating these trends across hospital levels are presented in S2 Fig.

### Postoperative complications and follow-up

Surgical wound infections were estimated to occur in less than 3% of cases by 71.6% (n = 489) of respondents, while 26.2% (n = 179) reported infection rates of 3–10%, and a smaller group (2.2%, n = 15) indicated rates between 10–15%. Endometritis was another noted concern, with 70.9% (n = 475) of respondents reporting a low incidence of less than 1%, 25.4% (n = 170) estimating rates of 1–3%, and 3.7% (n = 25) reporting rates of 3–5%. Wound dehiscence was also reported, with 89.6% (n = 588) of respondents stating an occurrence rate of less than 5%. In contrast, 9.9% (n = 65) reported rates of 5–10%, and 0.5% (n = 3) noted higher rates between 10–20% (Table 4). The data on these trends across hospital levels are detailed in S4 Table.

Regarding the hospital stay following a complicated CS, respondents report that the majority of patients (67.5%) remain hospitalized for 3–4 days, while a smaller proportion (9.3%, n = 69) require hospitalization for more than 5 days. Key follow-up measures include monitoring for significant blood loss during labor and the postpartum period (>1500 ml), which was ranked as the most important follow-up action by 80.0% of respondents. This is followed by infection prevention, ranked second by 66.3% of respondents. Lastly, the increased risk in future pregnancies was ranked in third position by most respondents (70.4%). Additionally, 14.4% (n = 107) of participants reported that a follow-up ultrasound was conducted to assess wound healing. The availability of ERAS/ACIERTO protocols for postoperative recovery was reported by 25.8% (n = 192) of respondents (Table 5).

**Table 5:** Follow-up after cesarean section reported in the national survey of obstetric protocols and outcomes in Spain (March-June 2024). n = 744.

Survey Question	Results		
**Hospitalization duration after complicated cesarean section, n (%)**			
3 days	241 (32.4)		
4 days	261 (35.1)		
5 days	173 (23.3)		
>5 days	69 (9.3)		
**Priority ranking of post-cesarean follow-up actions and controls, n (%)**	**1** ^**st**^	**2** ^**nd**^	**3** ^**rd**^
Blood loss during labor and postpartum (>1,500 ml)	595 (80.0)	106 (14.2)	43 (5.8)
Infection prevention	74 (9.9)	493 (66.3)	177 (23.8)
Prevention of future pregnancy risks	75 (10.1)	145 (19.5)	524 (70.4)
**Additional follow-up procedures after cesarean section, n (%)**			
Ultrasound to assess wound healing	107 (14.4)		
Availability or aplicationp of ERAS/ACIERTO protocol	192 (25.8)		

The average hospital stay for complicated CSs was around four days across all levels, with level 3 and level 4 hospitals reporting more extended stays beyond five days. Follow-up protocols varied, with level 4 hospitals using ERAS/ACIERTO protocols most frequently (32.3%) but performing fewer follow-up ultrasound assessments (6.2%) compared to level 1 hospitals (25.8%) (S5 Table).

## Discussion

This study highlights the evolving trends in managing complications during and after cesarean deliveries in Spain, particularly regarding perioperative protocols and intraoperative complications. The data reveal that intraoperative bleeding is among the most frequently reported challenges, followed by other critical complications such as difficult fetal extraction, uterine atony, and trauma to adjacent organs. Despite these risks, only a small proportion of hospitals reported having written preoperative protocols for complicated CSs, and less than a third reported formal simulation training or structured collaboration with other specialists such as urologists or interventional radiologists. In the context of globally increasing CS rates [15], addressing perioperative challenges through evidence-based strategies becomes increasingly important to reduce maternal morbidity, enhance intraoperative safety, and improve postoperative recovery.

In addition, the results reveal a notable division in the definition of what constitutes a complicated CS and in the perceptions of the importance attributed to the consequences of CSs. Approximately half of the respondents do not believe that the risks and implications of CSs are adequately emphasized, indicating variability in awareness or agreement on these issues. This is consistent with concerns raised by clinicians in other global studies, as summarized in the qualitative evidence synthesis published by Johansson et al. (2023) [16]. In that synthesis, CSs, particularly those without medical indication, were perceived to carry higher health risks compared to vaginal delivery. Despite this awareness, the increasing prevalence of non-medically indicated CS procedures suggests a disconnect between recognizing risks and addressing them effectively in clinical practice. As also noted in Johansson et al.‘s findings, clinicians emphasize the importance of incorporating comprehensive risk assessments into the decision-making process and ensuring these are clearly communicated to patients.

The findings also underscore the importance of establishing comprehensive protocols for complicated CSs. A total of 14.5% of respondents indicated that their hospitals have a written protocol for preoperative planning when a complicated cesarean section is anticipated. Among these institutions, 66.7% reported improvements following the implementation of the protocol. As highlighted in previous publications, measures such as an effective hemorrhage control during a complicated CS are critical; therefore, protocols should prioritize preventive strategies for managing intraoperative bleeding [11]. The results from the conducted survey indicate that around 41% and 35% of respondents reported that 1–5% and 5–10% of CSs that ultimately became complicated were not preoperatively identified as high-risk cases, respectively. It has been demonstrated that by implementing standardized protocols enables healthcare teams to proactively prepare for and manage high-risk cases [17]. Early identification, diagnostic imaging, blood product reservation, and prior specialist consultations (e.g., with urologists or interventional radiologists) could be systematically incorporated to improve outcomes and minimize risks. Hospitals already utilizing such protocols reported increased multidisciplinary involvement, particularly in hospitals where protocols facilitated collaboration with general surgeons and anesthesiologists, which improved patient outcomes [18,19].

As the number of repeat CSs increases, so does the associated maternal morbidity. In this study, respondents reported a median of 20% of all CSs performed classified as complicated. Studies highlight that a history of cesarean delivery is a risk factor for significant bleeding, particularly in cases of placenta previa, regardless of placental adherence [20]. Respondents of the present survey ranked previous abdominal surgeries as the most important factor regarding the difficulty in accessing the lower uterine segment. Jakobsson et al. (2013) [21] found that women with a prior CS are at increased risk for conditions such as placenta accreta, which is associated with higher rates of hemorrhage and the need for emergency hysterectomy. This is corroborated with additional studies showing a strong correlation between prior cesarean deliveries and complications like placenta previa, which can significantly exacerbate the risk of hemorrhage in subsequent deliveries [22]. Moreover, operational factors such as prolonged surgical time have been identified as independent risk factors for postpartum hemorrhage, particularly in women undergoing a second CS [23–25]. In this context, the present survey indicates that hemorrhage is an important concern, with more than 95% of respondents reporting the implementation of a blood product reserve when a complicated cesarean section is suspected, regardless of whether a written protocol is in place. They also reported that, in more than 35% of complicated CSs, bleeding of 1000–1500 ml occurs, and in nearly 6% of cases, bleeding exceeds 1500 mL. In fact, blood loss during labor and postpartum exceeding 1500 mL was reported by the surveyed gynecologists as the most frequent concern, highlighting this factor as one of the main aspects to be addressed in the management of complicated CS. These findings emphasize the importance of having standardized protocols and established procedures for managing complicated CSs, as well as proactive identification of potential complications. The absence of such protocols can lead to delays in the surgical process, which may exacerbate the risk of adverse maternal outcomes.

### Strengths and limitations

This study presents several strengths. First, it includes a large and diverse sample of 744 practicing gynecologists from various hospital levels across Spain, providing a comprehensive overview of current practices and perceptions regarding complicated cesarean sections. The stratification by hospital type and level allows for meaningful comparisons and insights into regional and institutional differences. Moreover, the use of a structured and detailed survey enabled the collection of nuanced information on both clinical protocols and real-world challenges in managing complicated cesarean deliveries.

However, the study also has some limitations. The sampling was based on voluntary participation from members of the Spanish Society of Gynecology and Obstetrics, which may introduce selection bias and limit generalizability to all obstetric care providers in Spain. The reliance on self-reported data may be subject to recall bias or social desirability bias, potentially affecting the accuracy of responses. Furthermore, the cross-sectional design captures practices and perceptions at a single point in time, limiting conclusions about causal relationships or temporal trends. Additionally, the survey focused primarily on clinical and procedural aspects but did not explore certain operational or structural factors, such as availability of institutional resources, administrative priorities, or staffing constraints, that could significantly influence the implementation of protocols. Finally, the survey was internally validated, providing a solid basis for data collection; even so, external validation in future studies could contribute to reinforcing its applicability across different clinical contexts.

## Conclusions

This national survey highlights that a limited proportion of hospitals in Spain have established written protocols for preoperative planning of complicated cesarean sections. The findings show frequent occurrences of significant intraoperative blood loss and common complications such as difficult fetal extraction, postpartum uterine atony, and organ trauma. Variability in protocol availability and multidisciplinary collaboration among hospitals indicates a need for greater standardization.

Based on these observations, the implementation of comprehensive, standardized protocols at the national level is strongly recommended, with a focus on early identification of high-risk cases, multidisciplinary teamwork, and effective hemorrhage management. In addition to their clinical relevance, the findings of this study offer valuable operational guidance for service providers, highlighting opportunities to strengthen institutional preparedness through the development of formal planning pathways, improved coordination between departments, and the integration of structured training strategies. The adoption of such measures is expected to reduce maternal morbidity, improve surgical and postoperative outcomes, and enhance overall patient safety and quality of care in complicated cesarean deliveries across Spain.

## Supporting information

S1 Table Characteristics of the respondents by hospital level (March-June 2024). n = 744. (DOCX)

S2 Table Characteristics of complicated cesarean sections by hospital level (March-June 2024). n = 744. (DOCX)

S3 Table Protocol of action by hospital level (March-June 2024). n = 744. (DOCX)

S4 Table Intraoperative and postoperative complications of cesarean section by hospital level (March-June 2024). n = 744. (DOCX)

S5 Table Follow-up after cesarean section by hospital level (March-June 2024). n = 744. (DOCX)

S1 Fig Geographical distribution of respondents by percentage across autonomous communities in Spain (March-June 2024). n = 744. (DOCX)

S2 Fig Ranking of factors in order of importance as reported by survey respondents regarding the difficulty in accessing the lower uterine segment (A), complicated fetal extraction in cesarean section (B), and anomalous placentation (C), stratified by hospital level (March-June 2024). The rankings are represented as percentages for each factor across different hospital levels. n = 744. PAS: placenta accreta spectrum.
(DOCX)
