# Supplementary material for: Complications in cesarean sections: A national survey of obstetric protocols and outcomes in Spain
Source: PLoS One. 2025 Sep 3;20(9):e0330352. doi: 10.1371/journal.pone.0330352 (PMC12407456; doi:10.1371/journal.pone.0330352)
Supplement: S1 Table — (DOCX) [file pone.0330352.s001.docx]

**Table S1.** Characteristics of the respondents by hospital level (March-June 2024). n=744.

| **Survey Question** | **Level 1 (n=124)** | **Level 2 (n=176)** | **Level 3 (n=248)** | **Level 4 (n=195)** |
| --- | --- | --- | --- | --- |
| **Age, median years (P25-P75)** | 42.0 (35.0-56.0) | 42.0 (35.0-50.0) | 41.0 (34.0-48.0) | 44.5 (35.0-55.0) |
| **Gender, n (%)** |  |  |  |  |
| Male | 31 (25.0) | 42 (23.9) | 49 (19.8) | 57 (29.2) |
| Female | 93 (75.0) | 134 (76.1) | 198 (80.2) | 138 (70.8) |
| **Experience as a gynecologist, median years (P25-P75)** | 18.2 (12.5) | 16.2 (10.4) | 15.5 (10.3) | 17.8 (10.7) |
| **Type of hospital, n (%)** |  |  |  |  |
| Public | 76 (61.3) | 124 (70.5) | 192 (77.4) | 166 (85.6) |
| Private | 32 (25.8) | 32 (18.2) | 29 (11.7) | 7 (3.6) |
| Public-private | 16 (12.9) | 20 (11.4) | 27 (10.9) | 21 (10.8) |
| **Cesarean sections per month, median (P25-P75)** | 8.0 (5.0-12.0) | 20.0 (14.0-30.0) | 30.0 (20.0-40.0) | 60.0 (40.0-100.0) |
| **Complicated cesarean sections per month, median (P25-P75)** | 1.0 (1.0-2.0) | 4.0 (2.0-5.0) | 5.0 (3.0-10.0) | 10.0 (5.0-20.0) |
| **Percentage of complicated cesarean sections per month, median (P25-P75)** | 16.7 (10.0-25.0) | 20.0 (10.0-31.8) | 20.0 (12.0-30.3) | 18.9 (10.0-31.3) |
