# Supplementary material for: Complications in cesarean sections: A national survey of obstetric protocols and outcomes in Spain
Source: PLoS One. 2025 Sep 3;20(9):e0330352. doi: 10.1371/journal.pone.0330352 (PMC12407456; doi:10.1371/journal.pone.0330352)
Supplement: S2 Table — (DOCX) [file pone.0330352.s002.docx]

**Table S2.** Characteristics of complicated cesarean sections by hospital level (March-June 2024). n=744.

| **Survey Question** | **Level 1 (n=124)** | **Level 2 (n=176)** | **Level 3 (n=248)** | **Level 4 (n=195)** |
| --- | --- | --- | --- | --- |
| **How would you define a complicated cesarean section? n (%)** |  |  |  |  |
| A cesarean section that may involve medical or surgical complexity (even if there are no subsequent complications) | 100 (80.6) | 138 (78.4) | 201 (81.0) | 156 (80.0) |
| The one that after its performance had more complications than usual (expected or not) | 69 (55.6) | 104 (59.1) | 154 (62.1) | 117 (60.0) |
| One that is expected to be performed by a senior attending physician | 6 (4.8) | 15 (8.5) | 26 (10.5) | 32 (16.4) |
| Other definitions | 3 (2.4) | 1 (0.6) | 4 (1.6) | 2 (1.0) |
| **Record of complicated cesarean sections and postoperative complications of obstetric surgery, n (%)** | 37 (29.8) | 38 (21.6) | 66 (26.6) | 56 (28.7) |
| **Are the consequences of cesarean section given the importance they deserve? n (%)** | 68 (54.8) | 88 (50.0) | 139 (56.0) | 94 (48.2) |
| **Frequency of complicated cesarean sections performed, n (%)** |  |  |  |  |
| Less than 20% | 111 (89.5) | 135 (76.7) | 164 (66.1) | 91 (46.7) |
| Between 20% and 30% | 11 (8.9) | 37 (21.0) | 70 (28.2) | 85 (43.6) |
| Between 30% and 40% | 2 (1.6) | 4 (2.3) | 14 (5.6) | 19 (9.7) |
| **Frequency of cesarean sections previously identified as potentially complicated, n (%)** |  |  |  |  |
| Less than 25% | 110 (88.7) | 148 (84.1) | 200 (80.6) | 141 (72.3) |
| Between 25% and 50% | 9 (7.3) | 20 (11.4) | 39 (15.7) | 46 (23.6) |
| More than 50% | 5 (4.0) | 8 (4.5) | 9 (3.6) | 8 (4.1) |
| **Frequency of complicated cesarean sections not previously identified as potentially complicated, n (%)** |  |  |  |  |
| 1-5% | 73 (58.9) | 74 (42.0) | 106 (42.7) | 54 (27.7) |
| 5-10% | 35 (28.2) | 72 (40.9) | 83 (33.5) | 70 (35.9) |
| 10-15% | 6 (4.8) | 12 (6.8) | 28 (11.3) | 32 (16.4) |
| 15-20% | 10 (8.1) | 18 (10.2) | 31 (12.5) | 39 (20.0) |
| **Type of incision chosen in a complicated cesarean section, n (%)** |  |  |  |  |
| Pfannensitiel | 89 (71.8) | 133 (75.6) | 196 (79.0) | 133 (68.2) |
| Misgav-Ladach | 32 (25.8) | 37 (21.0) | 43 (17.3) | 48 (24.6) |
| Median laparotomy | 3 (2.4) | 6 (3.4) | 9 (3.6) | 13 (6.7) |
| Cherney incision | 0 (0.0) | 0 (0.0) | 0 (0.0) | 1 (0.5) |
