# Supplementary material for: Complications in cesarean sections: A national survey of obstetric protocols and outcomes in Spain
Source: PLoS One. 2025 Sep 3;20(9):e0330352. doi: 10.1371/journal.pone.0330352 (PMC12407456; doi:10.1371/journal.pone.0330352)
Supplement: S3 Table — (DOCX) [file pone.0330352.s003.docx]

**Table S3.** Protocol of action by hospital level (March-June 2024). n=744.

| **Survey Question** | **Level 1 (n=124)** | | **Level 2 (n=176)** | **Level 3 (n=248)** | | | **Level 4 (n=195)** |  |
| --- | --- | --- | --- | --- | --- | --- | --- | --- |
| **Written protocol for preoperative planning in anticipation of a complicated cesarean section, n (%)** | | 16 (12.9) | 18 (10.2) | | | 30 (12.1) | 44 (22.6) | |
| **Actions in the event of a cesarean section that was not expected to be complicated, n (%)** | | 12 (75.0) | 11 (61.1) | | | 16 (53.3) | 32 (72.7) | |
| **Session or committee in which cases are presented, and strategies are developed, n (%)** | | 10 (62.5) | 14 (77.8) | | | 25 (83.3) | 38 (86.4) | |
| **Specialists decide who will be involved in an anticipated complicated cesarean section, n (%)** | |  |  | | |  |  | |
| Yes. Two attending physicians are required | | 9 (56.3) | 11 (61.1) | | | 18 (60.0) | 18 (45.0) | |
| It depends on the reason for the complexity | | 5 (31.3) | 5 (27.8) | | | 2 (6.7) | 0 (0.0) | |
| We do not decide who participates in the cesarean section | | 2 (12.5) | 2 (11.1) | | | 1 (3.3) | 3 (6.8) | |
| Yes. Two attending physicians are not required | | 0 (0.0) | 0 (0.0) | | | 1 (3.3) | 3 (6.8) | |
| **Prior contact with other specialists to evaluate the case, n (%)** | |  |  | | |  |  | |
| No contact with other specialists | | 3 (18.8) | 6 (33.3) | | | 10 (33.3) | 11 (25.0) | |
| General surgery | | 8 (50.0) | 7 (38.9) | | | 11 (36.7) | 24 (54.5) | |
| Urology | | 7 (43.8) | 8 (44.4) | | | 11 (36.7) | 31 (70.5) | |
| Interventional radiology | | 3 (18.8) | 4 (22.2) | | | 11 (36.7) | 24 (54.5) | |
| Depending on the case | | 0 (0.0) | 1 (5.6) | | | 1 (3.3) | 0 (0.0) | |
| Anesthesiologist | | 2 (12.5) | 2 (11.1) | | | 2 (6.7) | 1 (2.3) | |
| Other | | 3 (18.8) | 2 (11.1) | | | 0 (0.0) | 2 (4.5) | |
| **Actions implemented when a complicated cesarean section is suspected, n (%)** | |  |  | | |  |  | |
| Blood products reserve | | 16 (100.0) | 18 (100.0) | | | 29 (96.7) | 43 (97.7) | |
| Pre-anesthesia consultation | | 16 (100.0) | 18 (100.0) | | | 28 (93.3) | 43 (97.7) | |
| Consultation with other specialists | | 11 (68.8) | 12 (66.7) | | | 19 (63.3) | 37 (84.1) | |
| Diagnostic imaging methods | | 11 (68.8) | 8 (44.4) | | | 21 (70.0) | 32 (72.7) | |
| **Planned blood reserve, n (%)** | | 16 (100.0) | 18 (100.0) | | | 30 (100.0) | 43 (97.7) | |
| **Pre-training in complicated extractions with simulators, n (%)** | | 1 (6.3) | 8 (44.4) | | | 7 (23.3) | 23 (52.3) | |
| **Improvements observed following the implementation of the written protocol, n (%)** | | 7 (43.8) | 13 (72.2) | | | 20 (66.7) | 32 (72.7) | |
