# Supplementary material for: Complications in cesarean sections: A national survey of obstetric protocols and outcomes in Spain
Source: PLoS One. 2025 Sep 3;20(9):e0330352. doi: 10.1371/journal.pone.0330352 (PMC12407456; doi:10.1371/journal.pone.0330352)
Supplement: S4 Table — (DOCX) [file pone.0330352.s004.docx]

**Table S4.** Intraoperative and postoperative complications of cesarean section by hospital level (March-June 2024). n=744.

| **Survey Question** | **Level 1 (n=124)** | **Level 2 (n=176)** | **Level 3 (n=248)** | **Level 4 (n=195)** |
| --- | --- | --- | --- | --- |
| **Blood lost during a complicated cesarean section, n (%)** |  |  |  |  |
| <500 ml | 12 (9.7) | 18 (10.2) | 16 (6.5) | 13 (6.7) |
| 500-1000 ml | 64 (51.6) | 88 (50.0) | 129 (52.0) | 98 (50.3) |
| 1000-1500 ml | 45 (36.3) | 61 (34.7) | 86 (34.7) | 70 (35.9) |
| >1500 ml | 3 (2.4) | 9 (5.1) | 17 (6.9) | 14 (7.2) |
| **Other than hemorrhage, what other immediate complications are associated with a cesarean section in your practice? n (%)** |  |  |  |  |
| Difficult extraction of the fetus | 114 (91.9) | 159 (90.3) | 227 (91.5) | 174 (89.2) |
| Postpartum atony | 105 (84.7) | 139 (79.0) | 218 (87.9) | 172 (88.2) |
| Uterine tears | 91 (73.4) | 139 (79.0) | 200 (80.6) | 163 (83.6) |
| Trauma to adjacent organs | 49 (39.5) | 75 (42.6) | 122 (49.2) | 96 (49.2) |
| Other | 5 (4.0) | 3 (1.7) | 2 (0.8) | 5 (2.6) |
| **How often do complications arise in your hospital at cesarean section due to laceration or trauma to other organs? n (%)** |  |  |  |  |
| Urological injuries |  |  |  |  |
| Exceptional | 81 (65.3) | 91 (51.7) | 127 (51.2) | 94 (48.2) |
| 0.5-1% | 35 (28.2) | 61 (34.7) | 96 (38.7) | 84 (43.1) |
| 1-2% | 7 (5.6) | 21 (11.9) | 25 (10.1) | 13 (6.7) |
| >2% | 1 (0.8) | 3 (1.7) | 0 (0.0) | 4 (2.1) |
| Gastrointestinal lesions |  |  |  |  |
| Exceptional | 118 (95.2) | 161 (91.5) | 232 (93.5) | 179 (91.8) |
| 0.5-1% | 5 (4.0) | 14 (8.0) | 13 (5.2) | 13 (6.7) |
| 1-2% | 1 (0.8) | 1 (0.6) | 3 (1.2) | 3 (1.5) |
| >2% | 0 (0.0) | 0 (0.0) | 0 (0.0) | 0 (0.0) |
| **Complications after cesarean section, n (%)** |  |  |  |  |
| Surgical wound infections after cesarean section |  |  |  |  |
| <3% | 94 (81.0) | 124 (76.5) | 150 (66.4) | 120 (67.4) |
| 3-10% | 21 (18.1) | 36 (22.2) | 70 (31.0) | 52 (29.2) |
| 10-15% | 1 (0.9) | 2 (1.2) | 6 (2.7) | 6 (3.4) |
| Endometritis infections after cesarean section |  |  |  |  |
| <1% | 88 (77.9) | 120 (75.0) | 160 (71.4) | 107 (62.2) |
| 1-3% | 23 (20.4) | 34 (21.3) | 55 (24.6) | 57 (33.1) |
| 3-5% | 2 (1.8) | 6 (3.8) | 9 (4.0) | 8 (4.7) |
| Complications due to dehiscence after cesarean section |  |  |  |  |
| <5% | 99 (92.5) | 140 (89.2) | 194 (87.4) | 155 (91.2) |
| 5-10% | 8 (7.5) | 17 (10.8) | 27 (12.2) | 13 (7.6) |
| 10-20% | 0 (0.0) | 0 (0.0) | 1 (0.5) | 2 (1.2) |
