# Supplementary material for: Complications in cesarean sections: A national survey of obstetric protocols and outcomes in Spain
Source: PLoS One. 2025 Sep 3;20(9):e0330352. doi: 10.1371/journal.pone.0330352 (PMC12407456; doi:10.1371/journal.pone.0330352)
Supplement: S5 Table — (DOCX) [file pone.0330352.s005.docx]

**Table S5.** Follow-up after cesarean section by hospital level (March-June 2024). n=744.

| **Survey Question** | **Level 1 (n=124)** | | | **Level 2 (n=176)** | | | **Level 3 (n=248)** | | | **Level 4 (n=195)** | | |
| --- | --- | --- | --- | --- | --- | --- | --- | --- | --- | --- | --- | --- |
| **Hospitalization after complicated cesarean section, n (%)** |  | | |  | | |  | | |  | | |
| 3 days | 33 (26.6) | | | 64 (36.4) | | | 88 (35.5) | | | 56 (28.7) | | |
| 4 days | 49 (39.5) | | | 59 (33.5) | | | 88 (35.5) | | | 65 (33.3) | | |
| 5 days | 34 (27.4) | | | 42 (23.9) | | | 44 (17.7) | | | 53 (27.2) | | |
| >5 days | 8 (6.5) | | | 11 (6.3) | | | 28 (11.3) | | | 21 (10.8) | | |
| **Priority ranking of post-cesarean follow-up actions and controls, n (%)** | **1^st^** | **2^nd^** | **3^rd^** | **1^st^** | **2^nd^** | **3^rd^** | **1^st^** | **2^nd^** | **3^rd^** | **1^st^** | **2^nd^** | **3^rd^** |
| Blood loss during labor and postpartum (>1,500 ml) | 101 (81.5) | 19 (15.3) | 4 (3.2) | 134 (76.1) | 28 (15.9) | 14 (8.0) | 201 (81.0) | 31 (12.5) | 16 (6.5) | 158 (81.0) | 28 (14.4) | 9 (4.6) |
| Infection prevention | 9 (7.3) | 89 (71.8) | 26 (21.0) | 23 (13.1) | 110 (62.5) | 43 (24.4) | 24 (9.7) | 165 (66.5) | 59 (23.8) | 18 (9.2) | 129 (66.2) | 48 (24.6) |
| Increased risk in future pregnancies | 14 (11.3) | 16 (12.9) | 94 (75.8) | 19 (10.8) | 38 (21.6) | 119 (67.6) | 23 (9.3) | 52 (21.0) | 173 (69.8) | 19 (9.7) | 38 (19.5) | 138 (70.8) |
| **Subsequent ultrasound to see the state of wound healing, n (%)** | 32 (25.8) | | | 32 (18.2) | | | 31 (12.5) | | | 12 (6.2) | | |
| **Application or availability of ERAS/ACIERTO protocol in your department, n (%)** | 15 (12.1) | | | 42 (23.9) | | | 72 (29.0) | | | 63 (32.3) | | |
