# Supplementary material for: Complications in cesarean sections: A national survey of obstetric protocols and outcomes in Spain
Source: PLoS One. 2025 Sep 3;20(9):e0330352. doi: 10.1371/journal.pone.0330352 (PMC12407456; doi:10.1371/journal.pone.0330352)
Supplement: S1 Fig — (DOCX) [file pone.0330352.s006.docx]

**
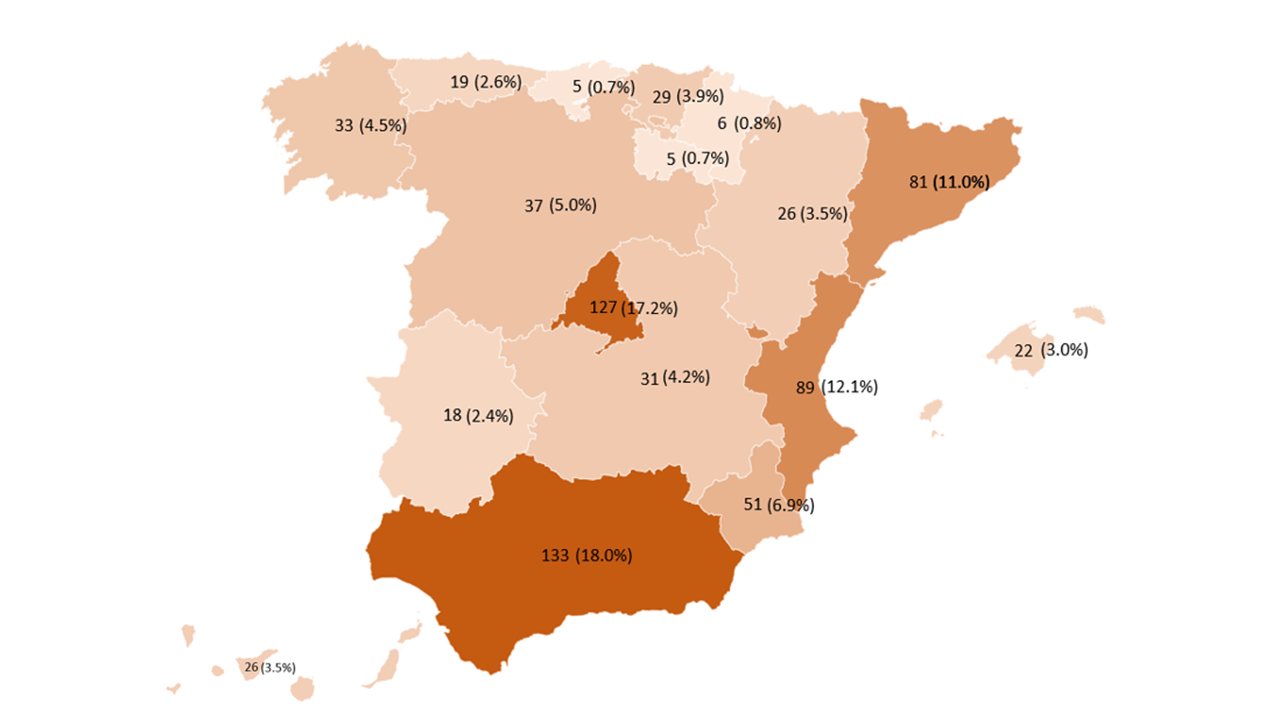
**

**Figure S1.** Geographical distribution of respondents by percentage across autonomous communities in Spain (March-June 2024). n=744. **The map was created using Excel’s built-in chart tool based solely on our own dataset. No external copyrighted content or proprietary map sources were used.*
