# Supplementary material for: Complications in cesarean sections: A national survey of obstetric protocols and outcomes in Spain
Source: PLoS One. 2025 Sep 3;20(9):e0330352. doi: 10.1371/journal.pone.0330352 (PMC12407456; doi:10.1371/journal.pone.0330352)
Supplement: S2 Fig — The rankings are represented as percentages for each factor across different hospital levels. n = 744. PAS: placenta accreta spectrum. (DOCX) [file pone.0330352.s007.docx]

**
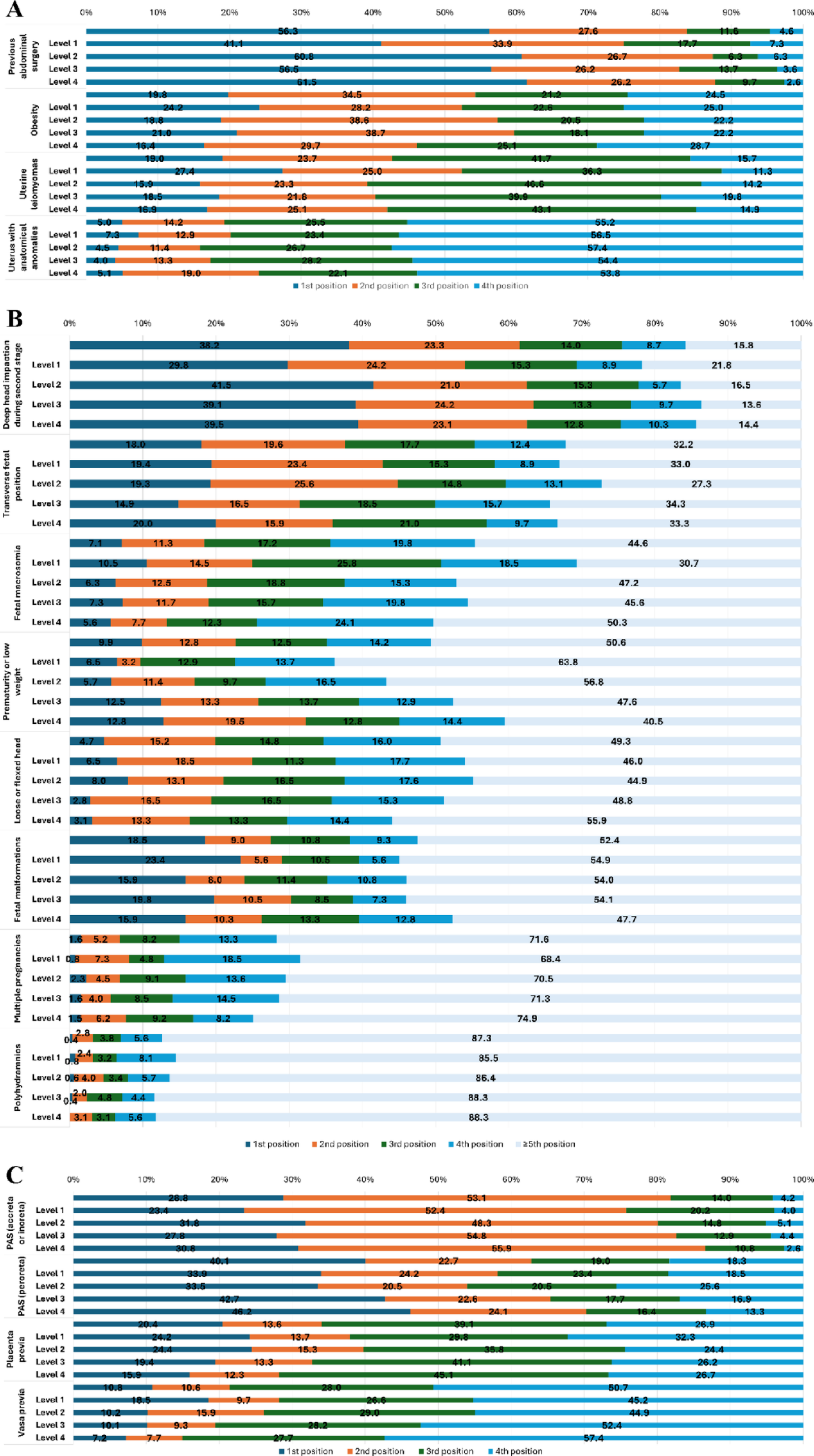
**

**Figure S2.** Ranking of factors in order of importance as reported by survey respondents regarding the difficulty in accessing the lower uterine segment (A), complicated fetal extraction in cesarean section (B), and anomalous placentation (C), stratified by hospital level (March-June 2024). The rankings are represented as percentages for each factor across different hospital levels. n=744. PAS: placenta accreta spectrum.
